# Hypoxia-Elicited Mesenchymal Stem Cell-Derived Small Extracellular Vesicles Alleviate Myocardial Infarction by Promoting Angiogenesis through the miR-214/Sufu Pathway

**DOI:** 10.1155/2023/1662182

**Published:** 2023-01-13

**Authors:** Lianbo Shao, Yihuan Chen, Jingjing Li, Jingfan Chao, Ziying Yang, Yinglong Ding, Han Shen, Yueqiu Chen, Zhenya Shen

**Affiliations:** Department of Cardiovascular Surgery of the First Affiliated Hospital & Institute for Cardiovascular Science, Soochow University, Suzhou, China

## Abstract

**Objective:**

Myocardial infarction is a leading cause of mortality worldwide. Angiogenesis in the infarct border zone is vital for heart function restoration after myocardial infarction. Hypoxia-induced MSC modification is a safe and effective approach for angiogenesis in clinical therapy; however, the mechanism still requires further investigation. In our study, we preconditioned human umbilical cord mesenchymal stem cells (huMSCs) with hypoxia and isolated the small extracellular vesicles (sEVs) to promote cardiac repair. We also investigated the potential mechanisms.

**Method:**

huMSCs were preconditioned with hypoxia (1% O_2_ and 5% CO_2_ at 37°C for 48 hours), and their sEVs were isolated using the Total Exosome Isolation reagent kit. To explore the role of miR-214 in MSC-derived sEVs, sEVs with low miR-214 expression were prepared by transfecting miR-214 inhibitor into huMSCs before hypoxia pretreatment. Scratch assays and tube formation assays were performed in sEVs cocultured with HUVECs to assess the proangiogenic capability of MSC-sEVs and MSC^hyp^-sEVs. Rat myocardial infarction models were used to investigate the ability of miR-214-differentially expressed sEVs in cardiac repair. Echocardiography, Masson's staining, and immunohistochemical staining for CD31 were performed to assess cardiac function, the ratio of myocardial fibrosis, and the capillary density after sEV implantation. The potential mechanism by which MSC^hyp^-sEVs enhance angiogenesis was explored *in vitro* by RT–qPCR and western blotting.

**Results:**

Tube formation and scratch assays demonstrated that the proangiogenic capability of huMSC-derived sEVs was enhanced by hypoxia pretreatment. Echocardiography and Masson's staining showed greater improvements in heart function and less ventricular remodeling after MSC^hyp^-sEV transplantation. The angiogenic capability was reduced following miR-214 knockdown in MSC^hyp^-sEVs. Furthermore, Sufu, a target of miR-214, was decreased, and hedgehog signaling was activated in HUVECs.

**Conclusion:**

We found that hypoxia induced miR-214 expression both in huMSCs and their sEVs. Transplantation of MSC^hyp^-sEVs into a myocardial infarction model improved cardiac repair by increasing angiogenesis. Mechanistically, MSC^hyp^-sEVs promote HUVEC tube formation and migration by transferring miR-214 into recipient cells, inhibiting Sufu expression, and activating the hedgehog pathway. Hypoxia-induced vesicle modification is a feasible way to restore heart function after myocardial infarction.

## 1. Introduction

Acute myocardial infarction (AMI) is a common cardiovascular disease with high morbidity and mortality worldwide [1]. The loss of cardiomyocytes caused by AMI leads to ventricular remodeling and the development of heart failure. Surgical and intervention therapy increased patient survival but failed to protect them from heart failure in the long term [2].

Stimulating endogenous repair in patients after MI is a therapeutic mechanism. Transplantation of mesenchymal stem cells (MSCs) into the ischemic myocardium has been used for cardiac repair, and the therapeutic effects are mainly due to the paracrine effects of small extracellular vesicles (sEVs), including exosomes [3]. Much evidence has demonstrated that sEVs play a functional role in delivering miRNAs to target cells in response to physiological changes or disease [4–7]. Compared with stem cells, sEVs not only maintain the functionality of their hosts but also confer several advantages, including lack of teratoma formation, low immunogenicity, and reduced biodegradability [8]. Growing evidence has demonstrated that sEVs sourced from MSCs have the potential to be a novel material for cardiac repair [3, 9, 10]. As the culture environment for the vesicle source cells changed, their contents were altered, and their biological effects were influenced as follows. Notably, MSC-derived vesicles exhibit distinct functions due to different oxygen concentrations in their culture microenvironments [11, 12]. It has been demonstrated that hypoxia treatment promotes the proangiogenic effects of MSC-derived vesicles and facilitates cardiac repair after AMI [13, 14].

miRNAs, as a class of posttranscriptional regulators, play multiple roles in the heart under physiological and pathological conditions. miR-214 plays an important regulatory role in cardiac repair, and its expression is altered in the myocardium after ischemia and upregulated in the plasma of patients with coronary artery disease [15, 16]. Upregulation of miR-214 protects against H_2_O_2_-induced apoptosis in cardiomyocytes, whereas knockdown of miR-214 aggravates cell loss after ischemic injury and causes deterioration of heart function [15, 17]. Previous studies have revealed that hypoxia induces the upregulation of miR-214 in various cell types [18–20]. However, the level of miR-214 in UMSC-derived vesicles induced by hypoxia and the mechanism of their proangiogenic effects due to miR-214 upregulation remain to be confirmed.

In this study, we compared the proangiogenic effects of sEVs derived from normoxia- or hypoxia-pretreated human umbilical cord MSCs (huMSCs) and further explored the possible associated proangiogenic mechanism.

## 2. Methods

### 2.1. Cell Culture and Hypoxia Pretreatment

huMSCs were kindly provided by the Stem Cell Bank, Chinese Academy of Sciences, cultured in DMEM:F12 medium with 10% exo-free FBS (precentrifuged at 110,000 × g for 16 hours to deplete exosomes in serum) in 21% O_2_ and 5% CO_2_ at 37°C. Hypoxia treatment was performed in a hypoxia chamber under 1% O_2_ and 5% CO_2_ at 37°C for 48 hours. The culture supernatant from hypoxia- and normoxia-induced huMSCs was collected for vesicle isolation.

### 2.2. MSC Flow Cytometry

At passage 3, huMSCs were digested with trypsin and stained with anti-human CD29-PE antibody, anti-human CD34-PE antibody, anti-human CD45-FITC antibody, anti-human CD73-PE antibody, anti-CD90-PE antibody, and anti-human HLA-DR antibody. After incubation at 4°C for 30 min protected from light, the cells were washed 3 times with PBS and then analyzed by flow cytometry (Millipore).

### 2.3. Small Extracellular Vesicle Isolation and Characterization

The conditioned medium was harvested and centrifuged at 2000 × g for 30 min to remove dead cells and cell debris and then filtered with a 0.22 *μ*m bore diameter filter to remove large vesicles. The supernatant was transferred into a new centrifuge tube, and 0.5 volumes of Total Exosome Isolation reagent (Life Technology) were added. The mixture was mixed into a homogenous solution, incubated at 4°C overnight, and then centrifuged at 10,000 × g for 1 hour at 4°C. Finally, vesicles were resuspended in a convenient volume of PBS and preserved at −80°C.

### 2.4. Transmission Electron Microscopy and NTA Identification of sEVs

It is hard to determine the purity and biogenesis pathway of the isolated particles. Therefore, we used the term of “sEVs” which is in accordance with the guidelines of International Society for Extracellular Vesicles (ISEV) [21, 22]. The isolated vesicles were resuspended in PBS, dropped onto a formvar-coated copper grid, and incubated for 30 min at room temperature. The grid was washed with PBS, and the samples were fixed in 2% paraformaldehyde for 10 min. Subsequently, the samples were washed with deionized water and then negatively stained with 2% uranyl acetate for 10 min. Finally, the samples were observed by a transmission electron microscope and photographed. The diameter distribution of the exosomes was analyzed with a NanoSight LM20 (NanoSight).

### 2.5. Extracellular Vesicle Uptake

Extracellular vesicles were labeled with PKH-26 (Sigma), a lipophilic dye that binds lipoproteins in a manner similar to phospholipids. The labeled vesicles were dissolved in EBM-2 medium at 100 *μ*g/mL and cocultured with HUVECs for 24 h. After incubation, HUVECs were fixed with 4% paraformaldehyde for 30 min, and the nuclei were stained with DAPI (2 *μ*g/mL) for 10 min. The cells were observed under a fluorescence microscope and photographed.

### 2.6. MI Model Induction and sEV Delivery

MI was induced in male Sprague–Dawley (SD) rats (260–280 g), and the preparation protocol was approved by the Ethics Committee of Soochow University. Animals were anesthetized with 2% isoflurane, and the neck and chest areas were shaved. Then, the rats were ventilated with a rodent ventilator by tracheal intubation. After exposing the heart, the left anterior descending coronary artery was ligated with a 6-0 nylon suture. Then, different vesicles (40 *μ*g suspended in 40 *μ*L PBS) or equal volumes of PBS were injected into the ischemic border zone at two different sites. Heart function was measured at 1, 7, 14, and 28 days post-MI using a Vevo 2100 ultrasound system (Visual Sonic). Left ventricular ejection fraction (LVEF) and fractional shortening (FS) were calculated and analyzed as previously described [23].

### 2.7. RT–qPCR Analysis

To detect the levels of miR-214 and its target-related genes, total RNA was extracted with TRIzol (Invitrogen), converted to cDNA (Takara), and analyzed using RT–qPCR. miR-214 levels from cells were normalized to small RNA U6, and levels from sEVs were normalized to cel-miR-39 that was added in the extraction process. The reverse transcription primers and detection primers were designed and obtained from RiboBio Co., Ltd. (Guangzhou). The mRNA levels of hedgehog signaling-related genes in cells were normalized GAPDH levels.

### 2.8. Knockdown of miR-214 in huMSC-Derived sEVs

To knock down the expression of miR-214, huMSCs were transfected with 50 nM miR-214 inhibitor using Lipo2000 according to the manufacturer's protocol. After transfection, huMSCs were cultured in DMEM:F12 with 10% exo-free FBS under hypoxia for 48 h, and the supernatant was harvested for vesicle isolation.

### 2.9. Matrigel Tube Formation Assay

Matrigel was mixed with basic medium at a 1 : 1 ratio, and 100 *μ*L of the mixture was added to each well of a 96-well plate and then incubated at 37°C for 30 min. HUVECs were resuspended in culture medium with different exosome (100 *μ*g/mL) treatments for 24 hours, and then, 5 × 10^4^ cells were seeded into 96-well plates for 6 hours. Tube formation was detected using an inverted microscope, and the tube length was quantified by the ImageJ software.

### 2.10. Dual-Luciferase Reporter Assay

The 3′UTR of the Sufu gene containing the predicted binding sequence for miR-214 was synthesized by an artificial chemical method and inserted into the psiCHECK™^−2^ luciferase reporter vector (Promega). The mutant binding sequence was inserted into the psiCHECK™^−2^ vector as a control. For the luciferase activity assay, 293T cells were seeded in a 24-well plate and cotransfected with 200 ng of the indicated psiCHECK™^−2^ vector, together with 200 nM miR-214 mimic. Forty-eight hours later, luciferase assays were performed. Relative luciferase activity was calculated as the ratio of firefly luminescence to Renilla luminescence.

### 2.11. Western Blot Analysis

To confirm the success of sEV isolation, in addition to the transmission electron microscopy and NTA, western blot analysis was also performed. EVs were quantified using a BCA Protein Assay Kit, boiled with an appropriate volume of 5x loading buffer, and subjected to separation by SDS-PAGE. The primary antibodies were incubated at 4°C overnight as follows: anti-CD63 antibody (1 : 2000; rabbit IgG, Abcam, ab134045) and anti-TSG101 antibody (1 : 1000; rabbit IgG, Abcam, ab125011). HUVECs were washed twice and lysed in RIPA buffer with proteinase inhibitor (1 mM PMSF) and running SDS-PAGE. The primary antibodies were incubated at 4°C overnight as follows: anti-Sufu antibody (1 : 1000; rabbit IgG, Abcam, ab259975), anti-Shh antibody (1 : 2000; rabbit IgG, Abcam ab53281), anti-Ptch antibody (1 : 1000; goat IgG, Abcam, ab109407), anti-VEGF antibody (1 : 1000; rabbit IgG, Abcam, ab214424), and anti-GAPDH antibody (1 : 3000; mouse IgG, CWBio, CW0100M). Horseradish peroxidase-conjugated corresponding secondary antibodies were incubated for another 1 hour at room temperature. Protein bands were detected using an ECL chemiluminescence kit (Meilun Bio).

### 2.12. Immunofluorescence Staining

Experimental rats were sacrificed after echocardiography detection at 28 days after MI. Heart samples were perfused with PBS, fixed in 4% paraformaldehyde, and embedded in paraffin; 5 *μ*m thick sections were cut for Masson's trichrome and immunohistochemistry staining to quantify the extent of fibrosis and infarct size. Capillary density was assessed and quantified using immunofluorescence for CD31 (1 : 100; Servicebio, GB11063-2).

### 2.13. Statistical and Data Analysis

All data are presented as mean ± SD, and two-tailed *t*-tests and one-way ANOVA were performed. *p* < 0.05 was considered significant.

## 3. Results

### 3.1. huMSC and sEV Identification

**Figure 1:**
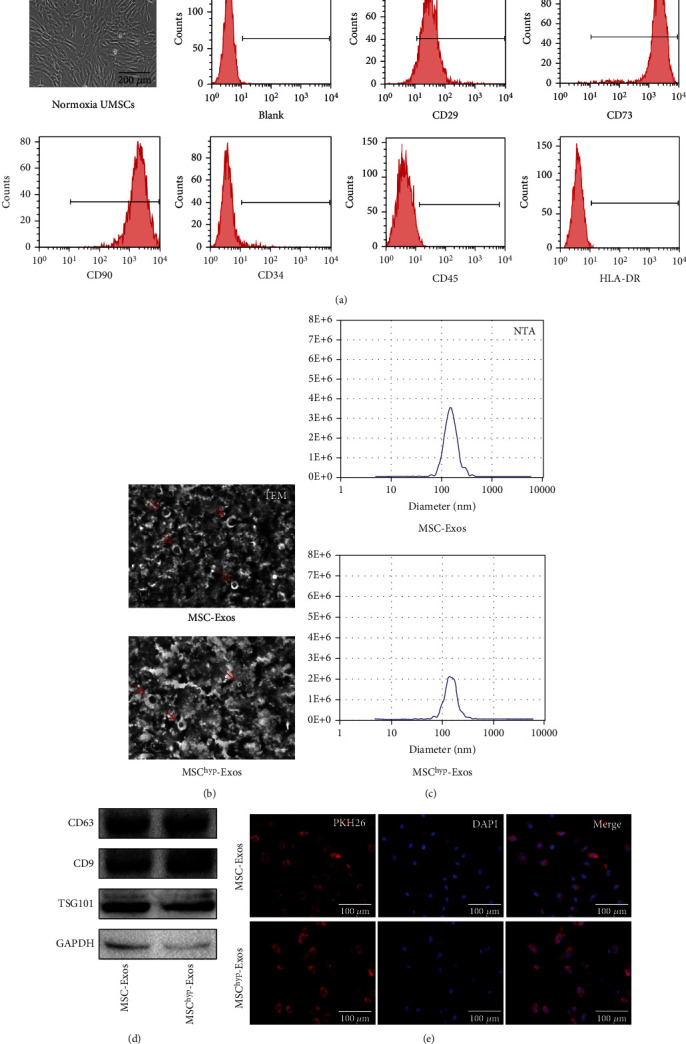
Characterization of huMSCs, sEVs, and endothelial cell cellular internalization. (a) Morphology and representative FCM characterization of huMSCs for typical surface antigens. (b) Transmission electron microscopy analysis was performed to identify the morphology of sEVs isolated from huMSCs with or without hypoxia treatment. Both of the two types vesicles were presented cup-shaped morphology (indicated by red arrows, bar = 200 nm). (c) NTA was applied to assess the size distribution of purified vesicles. (d) Western blotting analysis showed CD63, CD9, and TSG101 expressed in sEVs. (e) Fluorescence photomicrographs showed that DiI-labeled sEVs (red) were internalized into HUVECs after incubated for 24 h (100 *μ*g/mL, bar = 100 *μ*m).

huMSCs at passages 3-6 were typically spindle-shaped, and there was no significant difference in appearance with or without hypoxia induction for 48 hours. The identity of the MSCs was further analyzed by immunophenotypic staining for their antigen surface markers. The results showed that the cells were positive for CD29, CD73, and CD90 (positivity rate > 95%) and negative for CD34, CD45, and HLA–DR (negativity rate < 2%; Figure 1(a)). Hypoxia did not influence the expression of these markers (Suppl Figure 1).

To investigate the effects of sEVs on angiogenesis, we isolated vesicles sourced from normoxia-cultured huMSCs (MSC-sEVs) and hypoxia-induced huMSCs (MSC^hyp^-sEVs). The results of transmission electron microscopy (TEM) analysis showed that MSC-sEVs and MSC^hyp^-sEVs both presented a typical cup-shaped structure (Figure 1(b)). NTA data showed that the size distribution of the two types of vesicles was approximately 130 nm (Figure 1(c)). Western blotting also showed that both normoxic- and hypoxic-derived vesicles expressed CD63 and TSG101 (Figure 1(d)). All of these data indicated that hypoxia did not change the morphology of sEVs. To evaluate the internalization of vesicles by endothelial cells, sEVs were labeled with PKH26 and cocultured with HUVECs for 24 hours. Fluorescence images showed that vesicles were efficiently internalized into HUVECs, and there was no significant difference between the two types of vesicles (Figure 1(e)).

### 3.2. MSC^hyp^-sEVs Enhance Tube Formation and Migration of HUVECs

**Figure 2:**
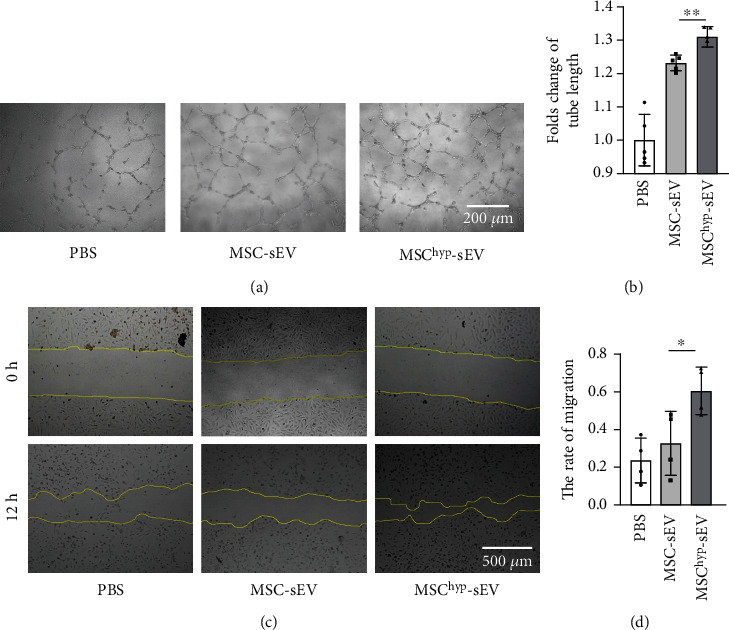
MSC^hyp^-sEVs promoted tube formation and migration of HUVECs. (a) Representative images showing the tube formation in HUVECs after cocultured with MSC-sEVs and MSC^hyp^-sEVs (bar = 200 *μ*m). (b) Quantification of tube length in different groups (*n* = 5). (c) The migration of HUVECs was detected at 12 hours after the scratch with MSC-sEVs and MSC^hyp^-sEV pretreatment (scale bar = 500 *μ*m). (d) Quantification of migration rate in different groups (*n* = 5). ^∗∗^*p* < 0.01.

The promotion of angiogenesis by MSC-derived vesicles is a well-documented mechanism underlying the therapeutic benefits of stem cells [24, 25]. To compare the angiogenic potential of these vesicles with or without hypoxia treatment, HUVEC tube formation and migration were examined *in vitro*. Consistent with previously published literature, our results showed that the formative tube length was increased upon treatment with sEVs compared with the control [26]. Additionally, the vessel length was longer with hypoxic sEV treatment than with normoxic sEV treatment (Figures 2(a) and 2(b)). The effect of MSC-sEVs on HUVEC migration was also investigated using a scratch assay. The migration rate of HUVECs treated with MSC^hyp^-sEVs was 1.8-fold greater than that of HUVECs treated with normoxic vesicles at 12 hours post scratch (Figures 2(c) and 2(d)). These data provide compelling evidence that MSC^hyp^-sEVs had stronger proangiogenic effects than MSC-sEVs *in vitro*.

### 3.3. Hypoxia-Induced sEVs Are More Effective in Cardiac Restoration

**Figure 3:**
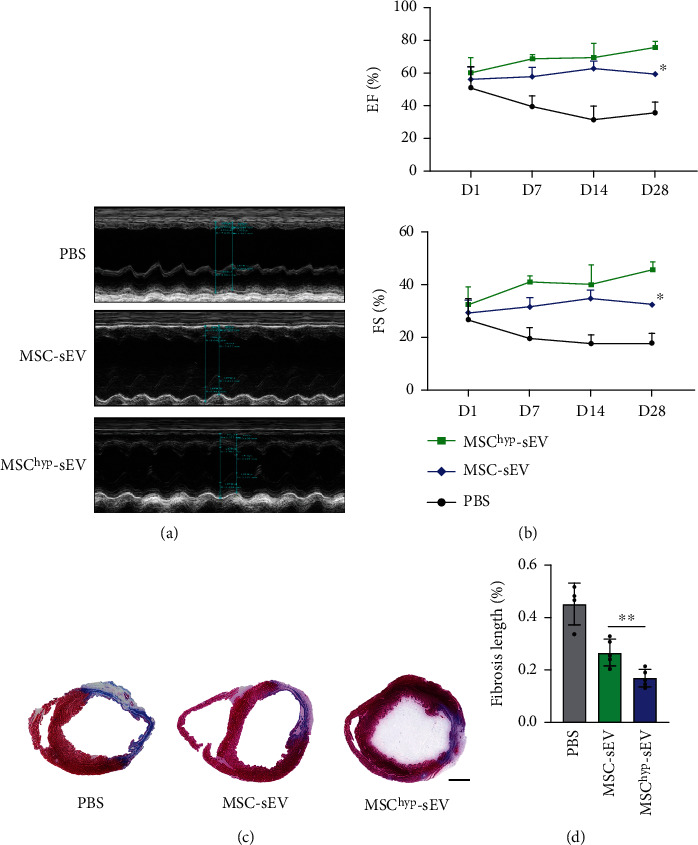
MSC^hyp^-sEV-enhanced heart function restores and ameliorated fibrosis after MI. (a) Representative echocardiogram of rat heart with MSC-sEVs and MSC^hyp^-sEV transplantation post-MI. (b) Significantly enhanced left ventricular function (EF and FS) in rats treated with MSC^hyp^-sEVs compared with other groups (*n* = 4-5 for each group). (c) Representative images of heart sections analyzed with Masson's staining at 28 days after LAD ligation (bar = 2 mm). (d) Quantitative analysis for the left ventricular fibrotic length (*n* = 4-5 for each group). ^∗^*p* < 0.05 and ^∗∗^*p* < 0.01.

To assess the cardiac protective ability of sEVs *in vivo*, PBS, MSC-sEVs, and MSC^hyp^-sEVs were transplanted into the infarcted myocardial border zone after LAD ligation. Echocardiography revealed that injection of both normoxic sEVs and hypoxic sEVs alleviated MI-induced cardiac dysfunction (Figures 3(a) and 3(b)). On day 1 after MI, there was no significant difference between these groups, and on day 7, the MSC-sEV group and MSC^hyp^-sEV group showed a moderate but not statistically significant improvement in EF and FS compared with the PBS group. The enhancement of EF and FS by MSC-sEVs or MSC^hyp^-sEVs was more significant on day 28 post-AMI (Figures 3(a) and 3(b)). Masson's staining showed significantly decreased fibrosis length (blue) in the MSC-sEV group (26.70 ± 5.66) and MSC^hyp^-sEV group (18.39 ± 4.74%) compared with the control group (45.09 ± 6.73%), and the fibrosis length in the MSC^hyp^-sEV group was even decreased compared with that in the MSC-sEV group (18.39 ± 4.74% vs. 26.70 ± 5.66, *n* = 4) (Figures 3(d) and 3(e)).

### 3.4. miR-214 Was Increased by Hypoxia Pretreatment and Targeted Sufu Expression

**Figure 4:**
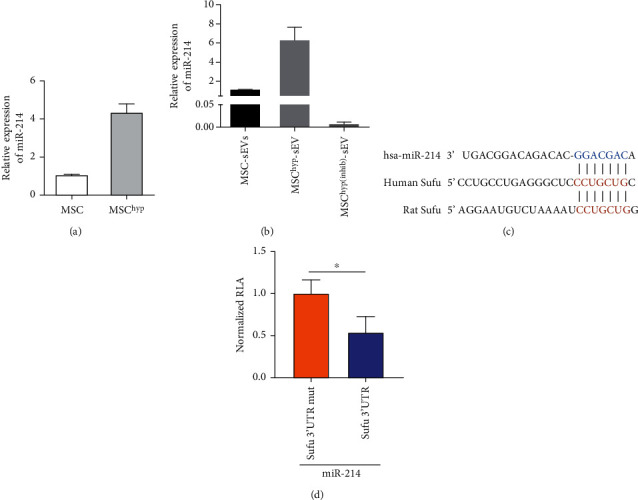
Hypoxia induces the expression of miR-214 and targets to Sufu. (a) RT-qPCR was applied to access miR-214 level in MSCs after normoxia or hypoxia preconditioning. (b) RT-qPCR was applied to access miR-214 level in sEVs after normoxia, hypoxia precondition, and miR-214 inhibitor transfection. (c) The putative miR-214 binding sequence in the 3′UTR of human Sufu and rat Sufu. (d) A luciferase reporter assay was performed to analyze the expression of Sufu with miR-214 transfection. ^∗∗^*p* < 0.01 and ^∗∗∗^*p* < 0.001.

Several previous studies have verified that miR-214 is upregulated with hypoxia treatment in several cell types [18, 20, 27]. However, whether it is expressed in huMSC-derived vesicles and the influence of hypoxia on its expression remain unknown. Therefore, we analyzed the level of miR-214 both in huMSCs and their derived vesicles by RT–PCR. The results showed that hypoxia induced miR-214 upregulation in huMSCs, and its expression was also upregulated in vesicles (6.22-fold) compared with the normoxia group (Figures 4(a) and 4(b)).

To further investigate the role of miR-214 in vesicles in angiogenesis, its putative targets were identified using miRTarBase. The results showed that Sufu, a key protein involved in the hedgehog pathway, is a putative target of miR-214 (Figure 4(c)). The sonic hedgehog pathway is an important regulator of angiogenesis. Sufu is a key regulator of signaling activation. To verify whether miR-214 regulates Sufu transcript levels, we transfected miR-214 mimics and a recombinant plasmid expressing luciferase driven by the 3′UTR of Sufu into 293T cells. The results of the luciferase assays showed that Sufu transcriptional activity decreased significantly following miR-214 mimic cotransfection (Figure 4(d)).

### 3.5. miR-214 Mediates MSC^hyp^-sEV Function in Promoting Angiogenesis

**Figure 5:**
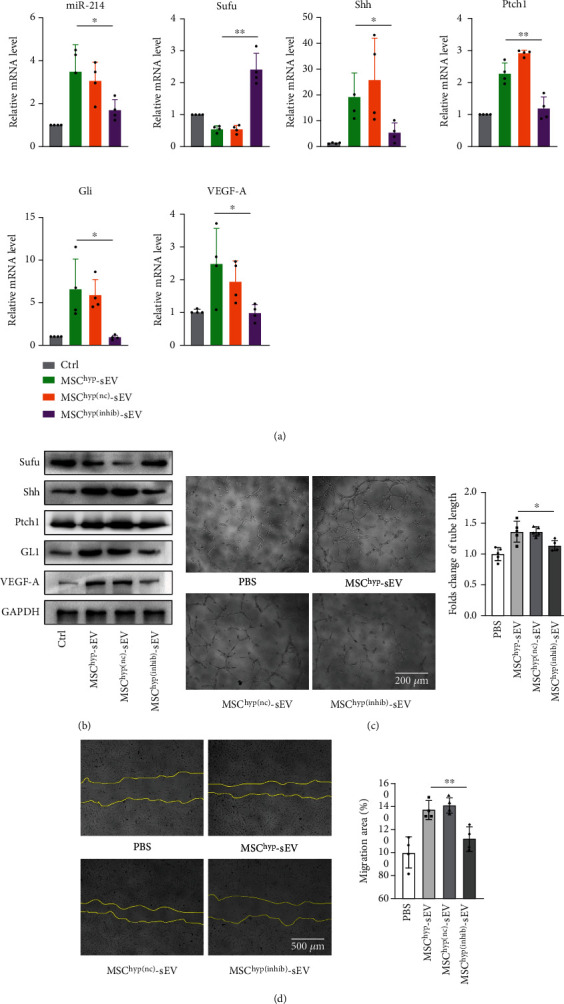
miR-214 and hedgehog pathway are involved in MSC^hyp^-sEV-mediated biological function of HUVECs. (a) Representative images of tube formation in HUVECs treated with PBS, MSC^hyp^-sEVs, MSC^hyp(nc)^-sEVs, and MSC^hyp(inhib)^-sEVs and quantification of tube length (*n* = 5). (b) Representative images of scratch assay in HUVECs treated with PBS, MSC^hyp^-sEVs, MSC^hyp(nc)^-sEVs, and MSC^hyp(inhib)^-sEVs and quantification of migration (*n* = 5, bar = 500 *μ*m). (c, d) RT-qPCR and western blot were performed to access hedgehog pathway-associated gene expression in HUVECs treated with PBS, MSC^hyp^-sEVs, MSC^hyp(nc)^-sEVs, and MSC^hyp(inhib)^-sEVs.

In classical hedgehog signaling, Sufu can bind with GLI and inhibit its translocation into the nucleus to mediate target gene transcription. In addition, the hedgehog pathway can regulate the expression of vascular endothelial growth factor (VEGF) in MSCs and cancer cells [28, 29]. Interestingly, we found that treating HUVECs with MSC^hyp^-sEVs activated hedgehog signaling and VEGF (Figure 5). Our dual-luciferase assay results showed that miR-214 inhibits Sufu expression. Furthermore, RT–qPCR and western blotting for Sufu were performed on RNA and protein, respectively, extracted from HUVECs after pretreatment with different sEVs. The results showed higher expression levels of miR-214 and lower expression levels of Sufu in HUVECs (Figure 5(a)). Other hedgehog pathway-related genes were also detected after coculture with vesicles differentially expressing miR-214. Compared with the control groups, notable Sufu downregulation was detected by RT–qPCR and western blot in the MSC^hyp^-sEV group. Moreover, mRNA and protein expression levels of Shh, Ptch, Gli, and VEGF-A were increased in the MSC^hyp^-sEV group. The reduced expression of miR-214 in sEVs also decreased the inhibitory effects of these genes (Figures 5(a) and 5(b)). Taken together, miR-214 activation inhibits Sufu expression and activates hedgehog signaling and VEGF in HUVECs.

We further explored the effects of miR-214-differentially expressed huMSC-derived sEVs on HUVEC tube formation capability and migration. According to the abovementioned results, we surmised that miR-214 may function as a mediator of the beneficial effects of huMSC vesicles after hypoxia treatment. To test our hypothesis, we silenced miR-214 expression in hypoxia-treated MSC-sEVs (Figure 3(b)). Compared with the MSC^hyp(nc)^-sEV (sEVs isolated from hypoxia-induced huMSCs transfected with miR-214 inhibitor negative control) group, miR-214-inhibited sEVs failed to improve tube formation and cell migration in HUVECs (Figures 5(c) and 5(d)).

### 3.6. miR-214 Mediates the Cardioprotective Function of MSC^hyp^-sEVs by Promoting Angiogenesis

**Figure 6:**
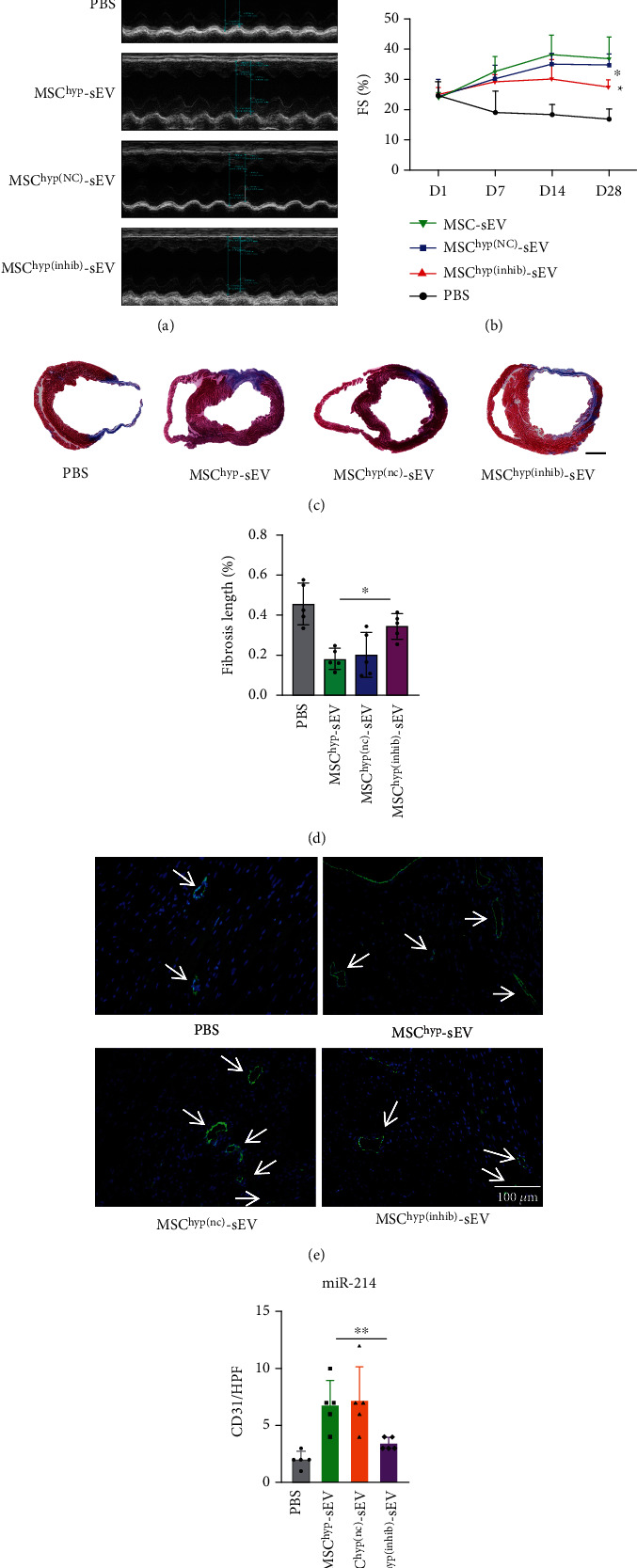
miR-214 is involved in MSC^hyp^-sEV-mediated improvement of myocardial repair. (a) Representative echocardiogram of the heart in rats transplanted with PBS, MSC^hyp^-sEVs, MSC^hyp(NC)^-sEVs, and MSC^hyp(inhib)^-sEVs on day 28 post-MI. (b) Rat heart LVEF and LVFE value on various time points of different groups. (c) Representative images of heart sections analyzed with Masson's staining (bar = 2 mm). (d) Quantitative data for the left ventricular fibrotic length. (e) CD31 immunofluorescence staining was performed to access angiogenesis at 4 weeks after MI and quantitative data for the capillary density (*n* = 5, bar = 100 *μ*m). ^∗^*p* < 0.05 and ^∗∗^*p* < 0.01.

To further investigate whether miR-214 mediates the cardioprotective effects of MSC^hyp^-sEVs *in vivo*, we delivered MSC^hyp^-sEVs, MSC^hyp(nc)^-sEVs, and MSC^hyp(inhit)^-sEVs by intramyocardial injection after LAD ligation. LVEF and FS were evaluated at various time points. The data revealed that the MSC^hyp^-sEV group and MSC^hyp(nc)^-sEV group showed similar levels of heart function; however, the MSC^hyp(inhib)^-sEV group showed significantly lower levels than these groups at 28-day post-MI (Figures 6(a)–6(d)). The infarct size after treatment with MSC^hyp(inhib)^-sEVs was not reduced compared with that after treatment with MSC^hyp^-sEVs and MSC^(nc)^-sEVs (Figure 6(d)).

To examine the angiogenetic effects conferred by sEV therapy on ischemic hearts, immunofluorescence for CD31 was performed to access capillary density. Based on the number of vessels per high power field (HPF) in the ischemic border, MSC-sEVs increased the capillary density compared with the control and MSC^hyp(inhib)^-sEV groups (Figures 6(e) and 6(f)). These results indicate that hypoxia pretreatment significantly enhanced the proangiogenic effects of huMSC-sEVs and promoted the functional restoration of ischemic hearts via the miR-214/Sufu pathway.

Taken together, these results indicate that hypoxia induces miR-214 expression in huMSC vesicles and promotes angiogenesis and cardiac repair at least partially through the hedgehog pathway.

## 4. Discussion

Although many treatments have been used to alleviate the initial cardiac injury during AMI, a novel therapeutic strategy is needed to decrease the subsequent development of cardiac remodeling and heart failure in the future. Stem cell transplantation is an effective method for injured cardiac repair; however, the inflammatory microenvironment post-MI makes it difficult for transplanted stem cells to survive. Emerging data have demonstrated that the cardioprotective function of stem cell transplantation mainly occurs via secreting paracrine factors, including sEVs [30]. When applied to ischemic cardiac disease therapy, sEVs not only exhibit therapeutic effects consistent with MSCs, such as reducing myocardial ischemia/reperfusion injury, but also have advantages of minimal immunogenicity and no teratoma formation. This suggested that sEV-based therapy holds great promise for cardiac repair [3, 31, 32].

Increasing evidence suggests that miRNAs in exosomes mediate the protective effects in cardiac repair [33–35]. However, other studies have indicated that exosomal miRNAs play limited roles in mediating the therapeutic functions [36–38]. After careful review of the relevant literatures, we found that the terms “exosomes” and “sEVs” both as the carrier of extracellular miRNAs are often confused. In several studies, the term “exosomes” most frequently refers to forms of miRNA transport involving many small extracellular vesicles. The vesicles prepared in our investigations with PEG precipitation may contain several distinct EV populations with high heterogeneity, including microvesicles, microparticles, and other similarly sized EVs; in accordance with the guidelines of ISEV, we used the term of “sEVs.” Comparing the classification of EVs based on size, density, preparation methods, and markers, defined exosomes as 100–200 nm particles with a density of 1.10–1.18 g/mL that carry exosome markers such as ALIX and TSG101, particularly exosomes having an endosomal formation from multivesicular bodies (MVBs) [37]. If the therapeutic purpose needs to be achieved by transferring miRNA, it is important to clearly define “exosomes” and “small extracellular vesicles (sEVs)” in studies on sEV-based therapy. Further, certain new markers that can identify the vesicles whether produced from MVB should be explored in future investigations.

**Figure 7:**
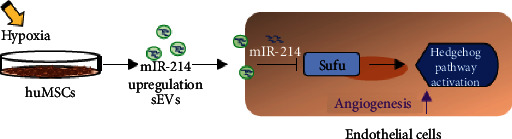
Effects and mechanisms of hypoxia pretreatment promote angiogenesis of huMSC-derived sEVs after myocardial infarction. Hypoxia pretreatment upregulates the expression level of miR-214 and increased miR-214 expression in the vesicles (MSC^hyp^-sEVs). MSC^hyp^-sEVs can be efficiently internalized by endothelial cells and activates hedgehog pathway resulting in modulation of angiogenesis.

The results described in our investigation demonstrated that (i) MSC^hyp^-sEVs enhanced endothelial cell tube formation and migration; (ii) hypoxia increased miR-214 expression both in huMSCs and their sEVs; (iii) MSC^hyp^-sEVs showed higher efficiency in restoring heart function and decreasing fibrosis area after AMI; and (iv) the protective effects of MSC^hyp^-sEVs might be partially mediated by the miR-214/Sufu pathway to stimulate angiogenesis in ischemic myocardium (Figure 7). To our knowledge, this is the first time that hypoxia-induced miR-214 expression was confirmed in huMSC-sEVs, which was consistent with the discovery by Wang et al. in bone marrow MSC-derived exosomes [39]; hence, we concluded that hypoxia-induced miR-214 expression is conserved in different MSCs.

Angiogenesis is a complex and highly regulated process to develop new blood vessels from existing vessels, including multiple steps such as endothelial cell activation, proliferation, and migration. Stimulating angiogenesis in the infarct zone can help salvage the myocardium for tissue regeneration [40]. Many investigations have shown that MSC-derived sEVs, including exosomes, can promote angiogenesis both *in vivo* and *in vitro* [41, 42]. Consistently, our results showed that huMSC-derived vesicles enhanced angiogenesis both with and without hypoxia preconditioning. Moreover, hypoxia pretreatment resulted in greater proangiogenic potential than normoxic pretreatment (Figure 2). Altogether, these findings suggest that promoting angiogenesis could be an important mechanism of stem cell-derived vesicles for cardiac repair.

It is well known that sEVs can mediate cellular communication under normal and pathological conditions by shuttling proteins, miRNAs, and lncRNAs into recipient cells [34]. Previous studies by us and others have shown that the modification of stem cells alters the imprint of their derived exosomes and has a superior effect on cardiac repair. Silencing B2M expression in MSCs upregulated exosomal miR-24 expression. The B2M-UMSC-derived exosomes conferred a better tissue-protective effect through the miR-24/Bim pathway both in AMI and hindlimb ischemic models [35, 43]. To enhance the cardioprotective capabilities of exosomes, Yu et al. overexpressed GATA4 in MSCs and found that the exosomes were enriched with antiapoptotic miRNAs [44]. Another study by Ma et al. showed that exosomes collected from Akt-overexpressing huMSCs enhanced their ability to augment angiogenesis and cardiac regeneration by activating PDGF [45]. Although these genetic approaches appear to be effective in increasing the cardioprotective effects by modifying the contents of MSC-derived sEVs, they are still infeasible in clinical practice due to unpredictable dangers. Compared with genetic modification, preconditioning huMSCs under hypoxia is a physical process, and it is safer and more clinically feasible.

In this study, our data showed that miR-214 was significantly increased both in MSCs and their vesicles under hypoxic preconditioning. Moreover, the expression level of Sufu was decreased, while hedgehog signaling was activated in HUVECs upon treatment with MSC^hyp^-sEVs. Inhibiting the expression of miR-214 in huMSC-sEVs reversed the expression of Sufu- and hedgehog-related genes in HUVECs after coculture. The hedgehog pathway is not only necessary for the development of the heart in the embryonic stage but also plays important roles in the formation of new coronary vessels in the heart [46]. After birth, the hedgehog pathway is gradually silenced in the heart; however, reactivating hedgehog signaling partially salvages ischemic damage in the heart and skeletal muscle by promoting injury-induced angiogenesis [47–49]. These results are in concordance with our results and further confirm that miR-214 promotes angiogenesis by targeting Sufu and activating the hedgehog pathway.

Nevertheless, some limitations existed in our investigations. There is no doubt that sEV transplantation is a promising therapeutic approach for cardiac repair, but the delivery routes should be considered. The intramyocardial injection is a highly effective route for sEV delivering and was performed in our study. However, because it requires open-heart surgery, it is difficult to be used for large-scale clinical applications. Intravenous injection is a suitable transplantation method for clinical usage, but it is difficult to deliver conventional sEVs into the injury site. Developing artificially modified sEVs which suitable for other clinical appropriated delivery routes, including intravenous injection, is necessary in future study.

The primary goal of cardiac repair is heart function restoration both in preclinical and clinical studies. Consistently, our results revealed a restoration of cardiac function in both the MSC-sEV- and MSC^hyp^-sEV-treated groups. Furthermore, hypoxia pretreatment is better for heart function recovery. Inhibiting miR-214 expression in sEVs impaired the proangiogenic capability. Correspondingly, the hedgehog pathway was crippled in HUVECs. All of these results showed that MSC-sourced vesicles promote angiogenesis partly through the miR-214/hedgehog pathway. However, the cardioprotective effects of miR-214 were greater than those of hedgehog signaling. Targets of miR-214, such as PTEN, Bcl2L11, and HIF1AN, also contributed to the observed protective effects of this miRNA in cardiac repair [15, 17, 50]. All of these results demonstrated that transient induction of miR-214 after AMI might provide a cardioprotective effect by inhibiting apoptosis and promoting angiogenesis.

## Data Availability

The data used to support the findings of this study are included within the article.

## Supplementary Materials

Supplementary Materials Supplementary Figure 1: characterization of hypoxia-induced huMSCs. (A) Morphology and representative FCM characterization of huMSCs for typical surface antigens.
