# Correction to “Hypoxia‐Elicited Mesenchymal Stem Cell‐Derived Small Extracellular Vesicles Alleviate Myocardial Infarction by Promoting Angiogenesis through the miR‐214/Sufu Pathway”

**DOI:** 10.1155/sci/9815781

**Published:** 2025-12-15

L. Shao, Y. Chen, J. Li, et al., “Hypoxia‐Elicited Mesenchymal Stem Cell‐Derived Small Extracellular Vesicles Alleviate Myocardial Infarction by Promoting Angiogenesis through the miR‐214/Sufu Pathway,” *Stem Cells International* 2023 (2023): 1662182, https://doi.org/10.1155/2023/1662182.

In the article titled “Hypoxia‐Elicited Mesenchymal Stem Cell‐Derived Small Extracellular Vesicles Alleviate Myocardial Infarction by Promoting Angiogenesis through the miR‐214/Sufu Pathway,” the panels b, c, d, and e of Figure 1 were labeled incorrectly.

More specifically:

“[MSC‐Exos]” should read “[MSC‐sEVs]”

and

“[MSChyp‐Exos]” should read “[MSChyp‐sEVs].”

The corrected Figure 1 is shown below:

Figure 1Characterization of huMSCs, sEVs, and endothelial cells cellular internalization.

**Figure:**
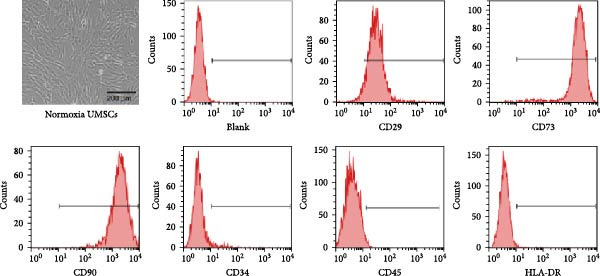
(a)

**Figure:**
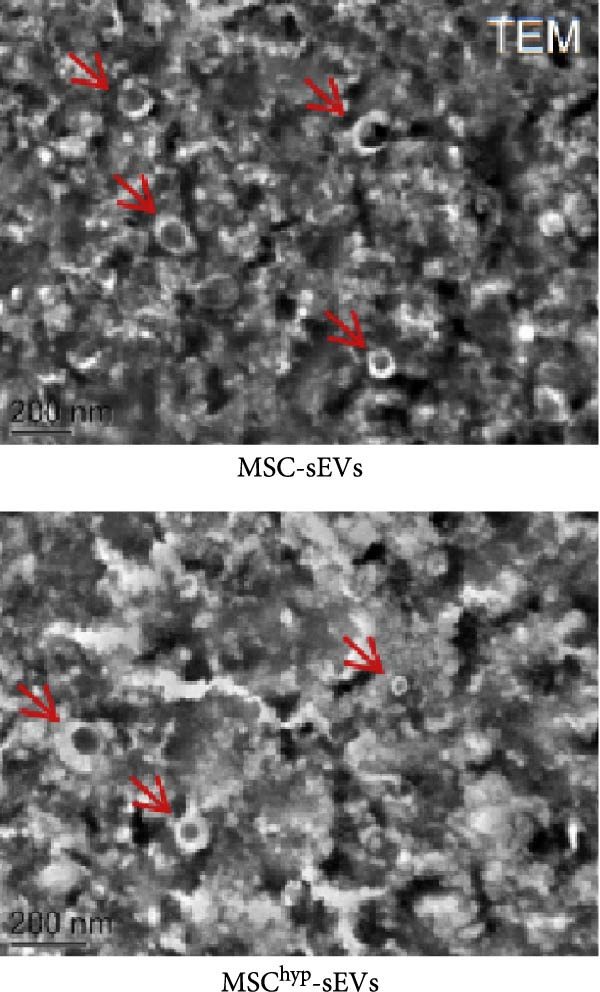
(b)

**Figure:**
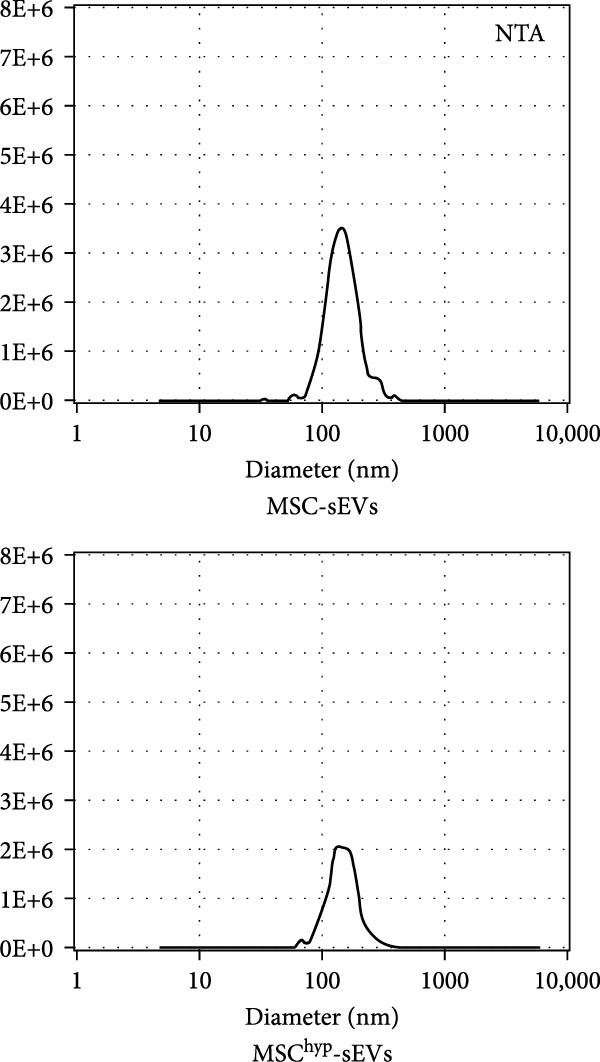
(c)

**Figure:**
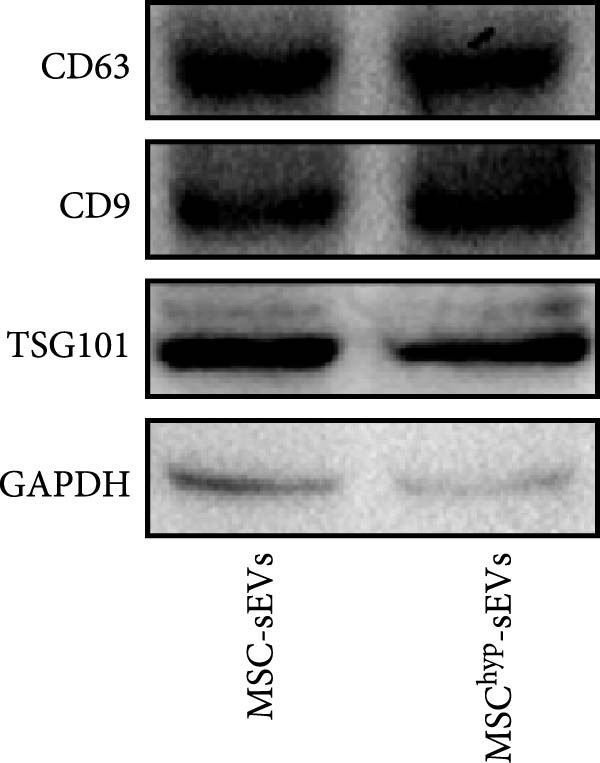
(d)

**Figure:**
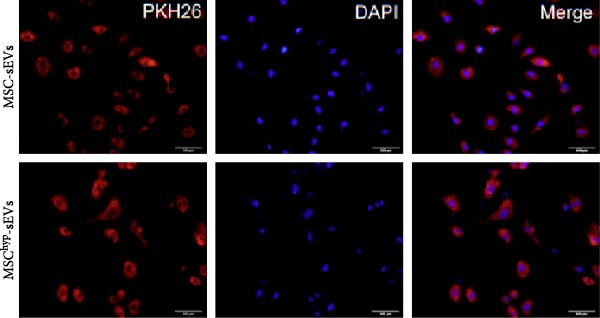
(e)

We apologize for this error.

