# Supplementary figures and images for: Hypoxia-Elicited Mesenchymal Stem Cell-Derived Small Extracellular Vesicles Alleviate Myocardial Infarction by Promoting Angiogenesis through the miR-214/Sufu Pathway
Source: Stem Cells Int. 2023 Jan 13;2023:1662182. doi: 10.1155/2023/1662182 (PMC11401710; doi:10.1155/2023/1662182)

A

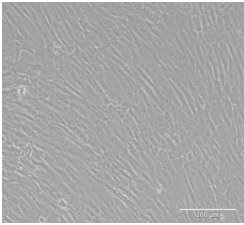

Hypoxia UMSCs

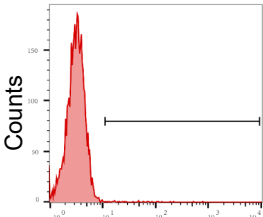

Blank

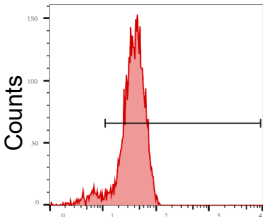

CD29

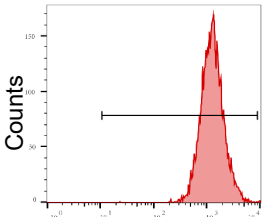

CD73

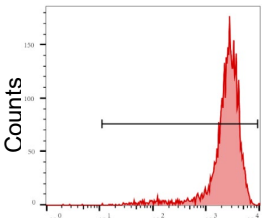

CD90

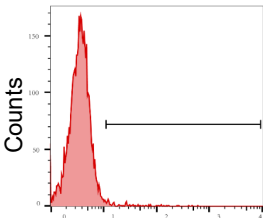

CD34

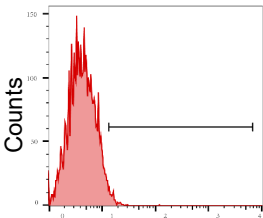

CD45

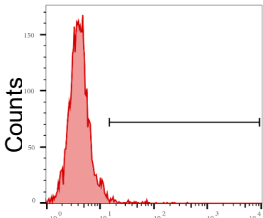

HLA-DR

Supplement: Supplementary Materials — Supplementary Figure 1: characterization of hypoxia-induced huMSCs. (A) Morphology and representative FCM characterization of huMSCs for typical surface antigens. [file 1662182.f1.pdf]
